# The activity of protein phosphatase 5 towards native clients is modulated by the middle- and C-terminal domains of Hsp90

**DOI:** 10.1038/srep17058

**Published:** 2015-11-23

**Authors:** Veronika Haslbeck, Julia M. Eckl, Adrian Drazic, Daniel A. Rutz, Oliver R. Lorenz, Kerstin Zimmermann, Thomas Kriehuber, Claudia Lindemann, Tobias Madl, Klaus Richter

**Affiliations:** 1 Center for Integrated Protein Science Munich at the Department Chemistry, Technische Universität München, Lichtenbergstraße 4, 85748 Garching, Germany; 2 Medical Proteome Center, Ruhr University Bochum, Universitätsstraße 150, 44780 Bochum, Germany; 3 Institute of Structural Biology, Helmholtz Zentrum München, 85764 Neuherberg, Germany; 4 Institute of Molecular Biology & Biochemistry, Center of Molecular Medicine, Medical University of Graz, 8010 Graz, Austria; 5 Omics Center Graz, BioTechMed Graz, 8010 Graz, Austria

## Abstract

Protein phosphatase 5 is involved in the regulation of kinases and transcription factors. The dephosphorylation activity is modulated by the molecular chaperone Hsp90, which binds to the TPR-domain of protein phosphatase 5. This interaction is dependent on the C-terminal MEEVD motif of Hsp90. We show that C-terminal Hsp90 fragments differ in their regulation of the phosphatase activity hinting to a more complex interaction. Also hydrodynamic parameters from analytical ultracentrifugation and small-angle X-ray scattering data suggest a compact structure for the Hsp90-protein phosphatase 5 complexes. Using crosslinking experiments coupled with mass spectrometric analysis and structural modelling we identify sites, which link the middle/C-terminal domain interface of *C. elegans* Hsp90 to the phosphatase domain of the corresponding kinase. Studying the relevance of the domains of Hsp90 for turnover of native substrates we find that ternary complexes with the glucocorticoid receptor (GR) are cooperatively formed by full-length Hsp90 and PPH-5. Our data suggest that the direct stimulation of the phosphatase activity by C-terminal Hsp90 fragments leads to increased dephosphorylation rates. These are further modulated by the binding of clients to the N-terminal and middle domain of Hsp90 and their presentation to the phosphatase within the phosphatase-Hsp90 complex.

Protein phosphatases are an integral part of cellular signalling networks. Based on their sequence, structure and catalytic mechanism they can be grouped into three different classes – phosphoprotein phosphatases (PPP), protein tyrosine phosphatases (PTP) and aspartate-based protein phosphatases. Protein phosphatase 5 (human: PP5) is a member of the PPP family with specificity for serine and threonine residues^1^,^2^,^3^. Sequence similarity compared to other members of this family such as PP1, PP2A and PP2B is high within the phosphatase domain^4^. But in contrast to the latter, PP5 contains three consecutive tetratricopeptide repeat (TPR) motifs located at its N-terminus and a C-terminal αJ subdomain^4^,^5^. An interaction between these two domains locks the enzyme in an auto inhibited state, limiting substrate entry to the active site of the phosphatase domain resulting in a low basal activity *in vitro*^1^,^5^,^6^,^7^,^8^.

The TPR domain is found in human PP5 and in its homologs from *Saccharomyces cerevisiae* (PPT1) and *Caenorhabditis elegans* (PPH-5), which share an overall identity of 40% (human versus yeast) and 60% (human versus *C. elegans*) (Blast Alignment). It is the known interaction site for heat shock protein 90 (Hsp90) and binds to the C-terminal MEEVD-peptide of Hsp90 with nanomolar to low micromolar affinity^9^,^10^. Binding of Hsp90 results in the release of intramolecular contacts between the TPR- and the αJ domain of the phosphatase, which abrogates the auto inhibition and facilitates access to the active site. In contrast to PP5, other PPP family members are modulated by the interaction with specific subunits, which control catalytic activity and substrate specificity of the holoenzyme^8^.

The protein phosphatase 5 is involved in many cellular functions including cell differentiation, migration, apoptosis, proliferation and DNA damage repair. Cell-lines from PP5 knock-out mice show an influence on the phosphorylation levels of p53 and the kinase Chk1^11^. Parts of its substrate spectrum consist of steroid hormone receptors and other transcription factors^4^ including various SMAD proteins, such as SMAD2 and SMAD4^12^. Likewise in nematodes SMA-4 (*C. elegans* homolog of SMAD-4) has been identified as interaction partner of PPH-5 in two-hybrid studies^13^. Clear evidence for an interaction of PP5 exists for the estrogen (ER) and the glucocorticoid receptor (GR), as early studies on proteins associated with GR have identified the phosphatase as part of Hsp90-GR complexes^14^. In this respect PP5 was also shown to affect the translocation of the hormone-activated receptor complex into the nucleus and to participate in the dephosphorylation of GR complexes *in vivo*^15^,^16^,^17^. Despite these insights, the mechanism of Hsp90-dependent activation of PP5 is unresolved to date.

Hsp90 consists of three domains – an N-terminal nucleotide binding domain, a middle domain relevant for client interaction and a C-terminal dimerization domain^18^,^19^,^20^. This structure is subject to conformational rearrangements, which are controlled by nucleotide binding and hydrolysis at the N-terminal domains of Hsp90^21^. Also Hsp90-cofactors, one of which is PP5, influence its conformations and its client specificity^22^,^23^. Here we obtained insight into structural and functional aspects of the protein complex between Hsp90/DAF-21 (sequence identity of human Hsp90β and *C. elegans* Hsp90: 76%) and protein phosphatase 5 from *C. elegans* (PPH-5) to understand the cooperation between these proteins during client processing^24^.

## Results

### CeHsp90 regulates PPH-5 also from outside the TPR-region

**Figure 1:**
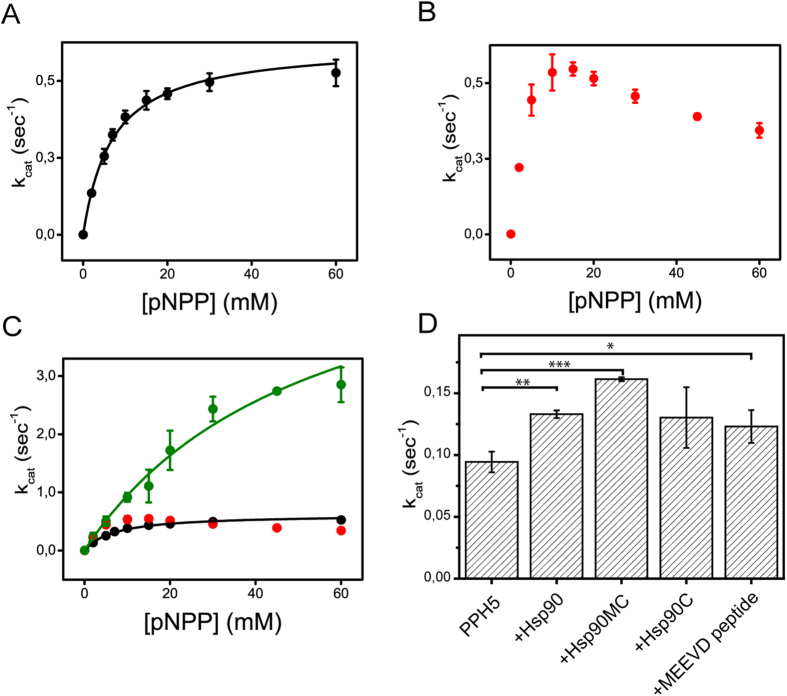
CeHsp90 influences the activity of PPH-5. (**A–C**) pNPP turnover was measured photometrically by recording the absorption at 410 nm. Shown is the dependence of the turnover on the substrate concentration. The maximal turnover (k_cat_) of PPH-5 was determined in absence (black) (**A**) and presence of either 3 μM full-length CeHsp90 (red) (**B**) or 60 μM of the CeHsp90-derived peptide AEEDASRMEEVD (green) (**C**). Results are expressed as mean ± SD (n ≥ 3). (**D**) 3 μM CeHsp90 constructs and 60 μM of the MEEVD-containing peptide influence the phosphatase activity (k_cat_) of PPH-5 towards the model peptide KRpTIRR (50 μM). The Hsp90 constructs alone had no effect on the model substrate. Results are expressed as mean ± SD (n ≥ 3). The significance was determined by Student’s t-test. Asterisks represent significant difference; p-values p ≤ 0.05 (*), p ≤ 0.01 (**), and p ≤ 0.001 (***).

Hsp90 activates protein phosphatase 5 by displacing the regulatory αJ helix of PP5, which is bound to the TPR-domain of the phosphatase in its auto inhibited state^6^. This depends on Hsp90’s C-terminal MEEVD peptide, which binds the TPR domain of PP5^6^,^25^. We analysed the enzymatic parameters of protein phosphatase 5 of *C. elegans* (PPH-5) in dephosphorylation assays using the substrate pNPP and recorded the influence of Hsp90 (Fig. 1). The K_M_-value of PPH-5 for pNPP was found to be 7.0 ± 0.4 mM (Fig. 1A). The specific activity of PPH-5 increased with rising amounts of substrate and reaches its maximum k_cat_ of 0.55 ± 0.09 s^−1^ at 60 mM pNPP. As expected from data of other eukaryotic systems, we observed an increase of the phosphatase activity in the presence of *C. elegans* Hsp90 (CeHsp90) (Fig. 1B), but this increase is considerably weaker compared to the homologous proteins and virtually absent at high substrate concentrations^6^. While the influence of CeHsp90 on v_max_ is very small in this system, the K_M_-value for pNPP is about fourfold reduced compared to the isolated PPH-5 protein. This influence on the K_M_-value suggests that CeHsp90 is also affecting substrate affinity, a characteristic which may require a more complex interaction between Hsp90 and PPH-5.

Thus, we tested whether similar stimulation properties can be observed by using the PPH-5 interacting peptide of CeHsp90. This MEEVD-containing peptide (AEEDASRMEEVD) can strongly activate PPH-5. Nevertheless, even at very high peptide concentrations (60 μM), an influence on the K_M_-value cannot be observed (Fig. 1C). Despite a strong stimulation of the dephosphorylating activity, leading to an almost 6-fold higher turnover, the K_M_-value is 18 mM versus the 2 mM in the presence of CeHsp90 (Fig. 1C). The MEEVD-containing peptide thus affects substrate turnover in a different manner compared to the full-length CeHsp90 protein. These results imply that the relationship between PPH-5 and CeHsp90 is more complex and suggest that beyond the primary interaction site between the MEEVD-peptide and the TPR-domain, further interaction sites may exist.

To address whether the activating influence of CeHsp90 on the phosphatase can also be observed with another PPH-5 substrate, we utilized the phosphorylated model peptide KRpTIRR and detected the cleavage of the phosphate group by a coupled enzymatic assay. Similar to pNPP, we observed an influence on the activity of PPH-5 in the presence of Hsp90, this time evident by a stimulation of activity by a factor of 1.4 from 0.094 ± 0.008 s^−1^ to 0.133 ± 0.003 s^−1^ (p ≤ 0.01). Omitting the N-terminal domain (Hsp90-MC) we obtained a slightly higher k_cat_ of 0.161 ± 0.002 s^−1^ (p ≤ 0.001). The isolated C-terminal Hsp90 fragment was not significantly able to stimulate the phosphatase and also the MEEVD-containing peptide (p ≤ 0.05) could only stimulate to a lower amount than Hsp90-MC (Fig. 1D). From these results we could expect that parts of the middle and C-terminal domain of Hsp90 (CeHsp90-MC) modulate the phosphatase activity in addition to the MEEVD peptide, which previously had been shown to comprise the primary interaction site between these two proteins^26^.

### Hsp90 complexes are formed with two PPH-5 molecules

**Figure 2:**
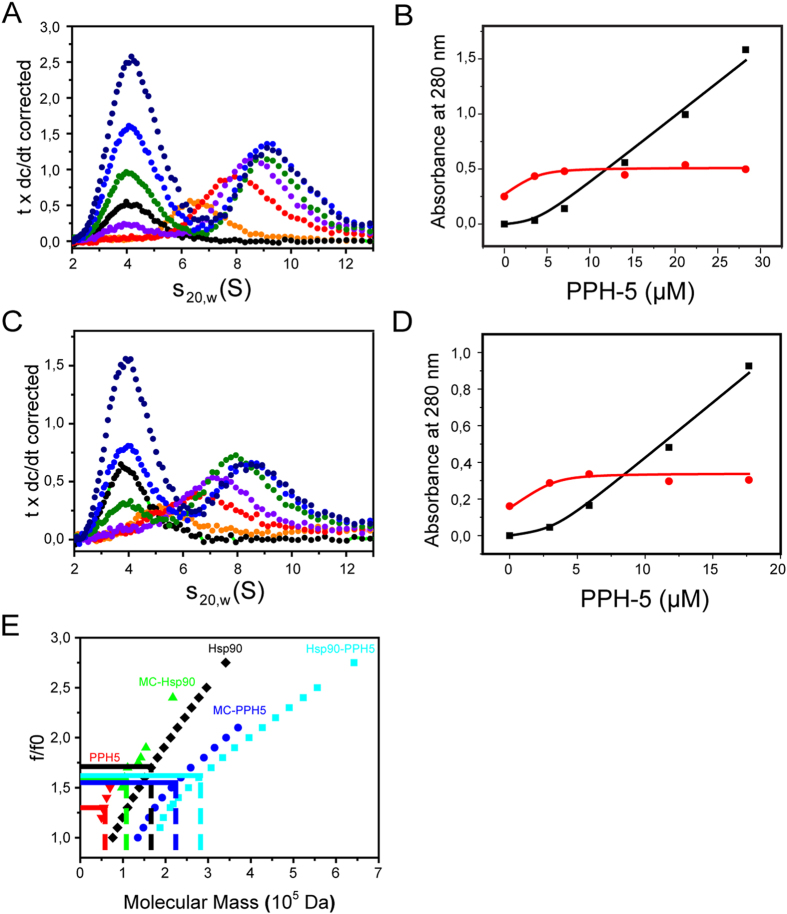
Stoichiometry of PPH-5-CeHsp90 complex formation. (**A**) The complex formation of PPH-5 and CeHsp90 was tested in a titration approach in analytical ultracentrifugation experiments. Shown are the dc/dt plots derived from Sedview from one full experimental setup. The absorption values were used to refine the concentrations in a global fit of the data. These concentrations were 4 μM CeHsp90 alone (orange), CeHsp90 +3.5 μM PPH-5 (red), +7 μM PPH-5 (violet), +14 μM PPH-5 (green), +21 μM PPH-5 (light blue), +28 μM PPH-5 (dark blue). As a control we used 7 μM PPH-5 alone (black) (**B**) Replot of the concentration values for free PPH-5 (black9 and CeHsp90 containing protein complexes (red) against added PPH-5. Data analysis as global fit of both curves was performed with the program Origin as described in the materials and methods section. (**C**) A similar titration approach as in (**A**) was performed for the MC-domain of CeHsp90. Titration of PPH-5 to 3.5 μM CeHsp90-MC (alone: orange, +1.4 μM PPH-5: red, +2.8 μM PPH-5: violet, +5.6 μM PPH-5: green, +12 μM PPH-5: blue, +16 μM PPH-5: marine blue) resulted in the formation of larger protein complexes. Shown again are the Sedview derived dc/dt-plots. 7 μM PPH-5 served as a control (black) (**D**) Replot of the concentration values for free PPH-5 (black) and in complex with CeHsp90-MC (red). Data analysis was performed as global fit of the concentrations of the individual species from the titration experiment. The red trace from (C) (3.5 μM CeHsp90-MC +1.4 μM PPH-5) was omitted as separation between the peaks could not be sufficiently observed. (**E**) Determination of the form factors using the stoichiometry obtained from the global fit and the differences in absorption for the sedimenting Hsp90 complexes. Form factors were determined for each of the following proteins or protein complexes: PPH-5 (red), CeHsp90 (black), CeHsp90-MC (green), PPH-5-CeHsp90 (blue), PPH-5-CeHsp90-MC (marine blue). The values for the plot of f/f0 against the molecular weight were obtained from Sedfit analysis of the experimental data set at a pre-set f/f0 parameter.

As indications towards a more complex regulation were uncovered, we tested whether additional interaction sites between phosphatase and Hsp90 can be observed during the formation of CeHsp90-PPH-5 complexes. We used analytical ultracentrifugation experiments to characterize the hydrodynamic properties, the stoichiometry and the affinity within the complexes of CeHsp90 and PPH-5. For this, different concentrations of PPH-5 were added to a constant concentration of CeHsp90 and the sedimentation properties were recorded to determine the distribution of assembled species. First, the stoichiometry of the saturated complex was determined based on the absorption signals. Full complex formation is obtained at PPH-5 concentrations above 15 μM (Fig. 2A). The initial absorption of dimeric CeHsp90 at 6.5 S (0.21 OD_280_) is increased to an OD_280_ of 0.37 sedimenting at 9.1 S (Fig. 2B). This increase in absorption reflects the binding of two molecules of PPH-5 to the CeHsp90 dimer. A global fit confirms the stoichiometry and suggests a binding affinity in the range of 1 μM for the first and second PPH-5 molecule, which is supported by the monitoring of substantial amounts of free PPH-5 at PPH-5 concentrations, in which CeHsp90-binding sites are not yet saturated. We performed a similar titration experiment with the MC domain of CeHsp90 (Fig. 2C). Here, the absorption of the CeHsp90-MC in the absence of PPH-5 was 0.19 sedimenting with 5.5 S. At full saturation the complex sedimented at 8.1 S with an absorption of 0.36, which reflects the binding of two PPH-5 molecules to the CeHsp90-MC dimer. This stoichiometry is also consistent with a global fit of the absorption values of CeHsp90 and free PPH-5 (Fig. 2D).

The very large shift in sedimentation coefficients may reflect a compaction or coverage of CeHsp90 surface areas by PPH-5 in the macromolecular assembly in addition to the mass increase upon binding of PPH-5. Given that the stoichiometry of the complex and the s_20,w_ coefficients are well described from these experiments we can determine the form factor (frictional coefficient) of the complex based on the last points in the titrations. We first used Hsp90, which can be assumed to have a molecular mass of ~166 kDa at the concentrations we used given that the dimerization constant of CeHsp90 is in the nanomolar range^21^. CeHsp90 alone sedimented with an s_20,w_ of 6.5 S. Restricting the molecular mass of the dimeric protein to 166 kDa, an f/f0-coefficient of 1.71+/−0.02 is obtained, which highlights the non-spherical character of Hsp90 as evident from crystal structures (Fig. 2E)^20^,^27^. The frictional coefficient is reduced to 1.60+/−0.02 upon binding of the two PPH-5 molecules. Likewise the f/f0 for CeHsp90-MC (1.61+/−0.02) is reduced to 1.55+/0.02 upon binding of the phosphatase cofactors. Isolated PPH-5, being a more spherical protein, is characterized by an f/f0 of 1.34+/−0.02 (Fig. 2E). These data show that the non-spherical character of the CeHsp90 scaffold is not further extended by binding of PPH-5 to CeHsp90’s flexible C-terminus. Instead the hydrodynamic properties of the heterotetrameric protein complex appear more sphere-like and compact compared to CeHsp90 itself. This might be due to a conformational rearrangement of Hsp90 in the complex, the coverage of a surface area in the central part of Hsp90, the binding of the long disordered Hsp90 C-terminus, or a combination thereof.

### SAXS implies a compacted complex form

To get further information on the complex formation and on the molecular details of the overall conformation of the Hsp90-PPH-5 complex, we recorded small-angle X-ray scattering (SAXS) data. In comparison to human Hsp90β^28^,^29^, SAXS data indicate that CeHsp90 adopts a more compact conformation with smaller maximal dimensions (D_max_ = 195 vs. 240 Å) and smaller radius of gyration (R_g_ = 59.1 vs. 64.2 Å) hinting to higher populations of closed states for CeHsp90 (Table 1). This is in agreement with recent biochemical studies on the hydrolysis mechanism of the nematode Hsp90 protein, which postulate also closer conformations^30^.

**Figure 3:**
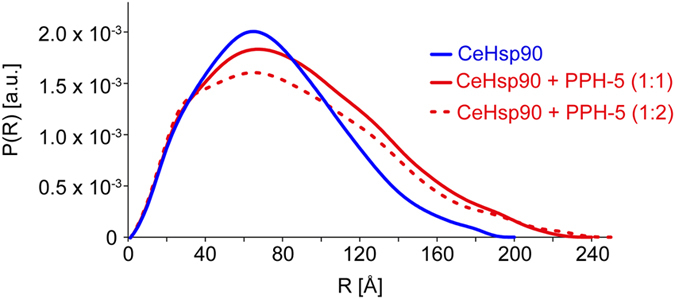
SAXS curves imply a small extension of the protein upon complex formation. SAXS data showing a comparison of the experimental radial density distributions of CeHsp90 (blue) and with increasing stoichiometric ratios of PPH-5 as indicated (red straight line and red dashed).

We then performed SAXS experiments on the individual proteins and on the CeHsp90-PPH-5 complex at different ratios between CeHsp90 and PPH-5. We found that the apparent molecular mass and R_g_ of the CeHsp90-PPH-5 complex increased for a 2:1 ratio (CeHsp90 monomer:PPH-5) (Fig. 3, Table 1) the apparent molecular weight of the proteins in solution is 240 kDa. Binding of PPH-5 to CeHsp90 also results in an increase of radial density at higher distances. Above that ratio, both the apparent molecular mass and the R_g_ decreased due to the accumulation of unbound PPH-5. This indicates that the two subunits of CeHsp90 bind one molecule of PPH-5 with sufficient affinity to obtain complex formation, while the binding of two PPH-5 molecules to one CeHsp90 dimer seems to occur together with substantial amounts of free PPH-5. This matches the AUC data, which suggest binding constants for the two PPH-5 molecules between 1 μM and 3 μM. While the species are separated in AUC, SAXS provides a population-weighted snapshot of all conformations in solution, making it impossible to obtain the pure saturated Hsp90_2_-PPH5_2_ complex in the absence of free PPH-5.

### Crosslinking and MS-analysis suggests a binding site at the Hsp90 M-C interface

**Figure 4:**
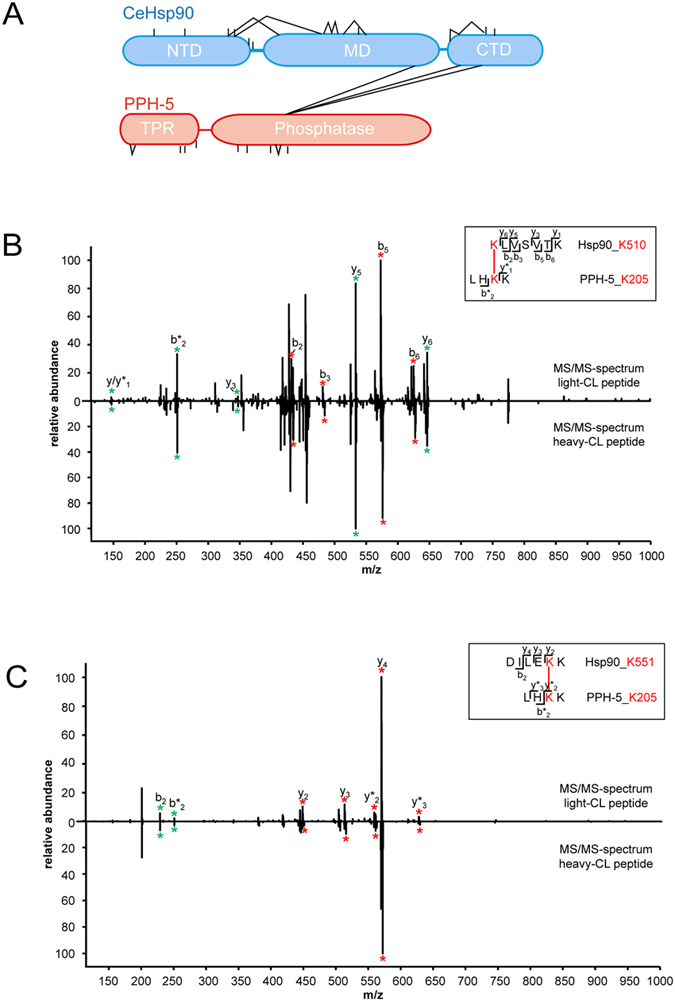
Crosslink- and mass spectrometric analysis of the Hsp90 PPH-5 interaction. MS/MS-spectra of the identified light and the corresponding heavy intermolecular cross-link peptide (**A**) Shown is a schematic representation of the results of three crosslinking experiments. Analysis revealed a potential contact site between the phosphatase domain of PPH-5 (red) and the middle/C-terminal interface of CeHsp90 (blue) as indicated by the lines connecting the two proteins. (**B**) CeHsp90-K510 – PPH-5-K205 and cross-link peptide (**C**) CeHsp90-K551 – PPH-5-K205, common b- and y-ions are marked with green stars. Cross-linker containing fragment-ions (red stars) of the light crosslinked peptide (light-CL peptide) and the heavy crosslinked peptide (heavy-CL peptide) are illustrated in the MS/MS-spectra by red stars and show a mass shift of 6.0437/z.

**Figure 5:**
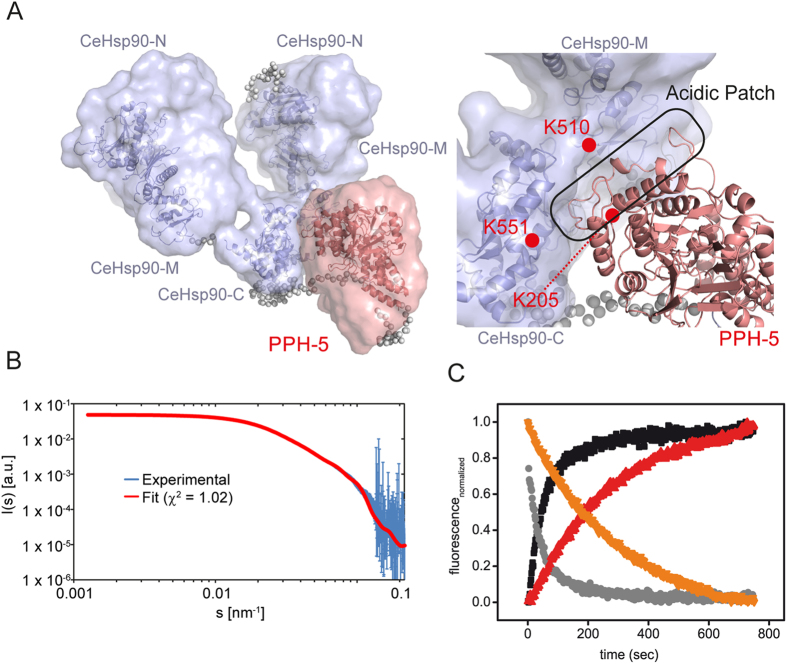
SAXS/MS structure calculations. (**A**) Structural model for the CeHsp90-PPH-5 complex. The surface represents the conformational space sampled in the 5 best structures according to the fit between the experimental and back-calculated SAXS data. Structures are aligned to the CeHsp90-C domain. Positions of the cross-linked lysine residues and of the acidic patch in PPH-5 are indicated. (**B**) Comparison of experimental CeHsp90-PPH-5 SAXS data with SAXS data back-calculated from the CORAL model of the CeHsp90-PPH-5 complex. Both, the s, and I(s) axes are shown in a logarithmic representation. The angular ranges from 0.0014–0.3 nm^−1^ are compared. (**C**) Subunit exchange experiment of yHsp90 in the absence and presence of PPH-5. A ten-fold excess of unlabelled yeast Hsp90 was added to an equal mixture of 0.2 μM of N-terminally ATTO488-labeled Hsp90 and 0.2 μM N-terminally ATTO550-labeled Hsp90 in absence and presence of PPH-5 (3 μM). The donor channel signal is depicted in absence of PPH-5 (black) and in presence of the phosphatase (red) as well as the acceptor channel signal (without PPH-5 – grey, with PPH-5 – orange).

To obtain a structural model for the complex, we set off to obtain further structural MS crosslinking data to combine with the SAXS data in an integrative approach. As hydrodynamic parameters indicate that the PPH-5 protein is not bent away from the CeHsp90 scaffold but could prefer a position, in which the PPH-5 molecule is attached to the CeHsp90 dimer we were interested to obtain information towards the positioning of secondary contact sites between the two proteins. Therefore, the two proteins were exposed to chemical crosslinking with the isotope-tagged crosslinker H_6_/D_6_-DSSG with subsequent identification of the crosslinked peptides. This approach has been applied before for different protein complexes and is able to report on potential contact sites in proteins, which can come close enough to allow chemical crosslinking^31^. Specific CeHsp90-PPH-5 crosslinking products representing the large protein complex were obtained from SDS-PAGE gels at the expected size. Bands were subjected to tryptic digestion and analysed by high-resolution mass spectrometry. Peptides containing the H_6_/D_6_-crosslinker were identified based on the characteristic 6.043 Da/z separation of the H_6_-DSSG and the D_6_-DSSG containing peptides. These peptides were confirmed based on the ion fragmentation patterns in MS/MS-spectra of the light (H_6_)- and corresponding heavy (D_6_) cross-linked peptide (Table 2). Most of the identified intramolecular crosslinked peptides contain an attached crosslinker molecule or connect nearby lysine residues in the same peptide chain (Fig. 4). Besides, the intramolecular crosslinks we also detected intermolecular crosslinks between PPH-5 and CeHsp90 reproducible in three performed experiments (Table 2). As such K510 of CeHsp90 is crosslinked to K205 of PPH-5 in all these experiments (Fig. 5A). Other intermolecular crosslinks reside also in this part of the protein, such as K551 to K205 (Fig. 4B) or K585 to K206 (Table 2). The amino acid positions of the crosslinked lysine residues highlight that in particular the M-C interface of CeHsp90 is prone to crosslinking with the N-terminal part of PPH-5’s phosphatase domain (Fig. 4C). We did not observe crosslinks between the C-terminal MEEVD-peptide and the TPR-domain, but this likely is due to the absence of lysine residues in the last 15 amino acids of CeHsp90. In general, the detection of these crosslinks shows that PPH-5 certainly has the capability to reach with its phosphatase domain toward the proposed client binding site of Hsp90 and thereby influence client binding and processing.

### Structural insights into CeHsp90 and PPH-5 interaction

To obtain a rigid body structural model of the CeHsp90-PPH-5 complex, we combined the SAXS data of the 2:1 complex and restraints from the mass spectrometric analysis with a homology model based on published crystal structures of Hsp90 (2CG9)^27^ and a recently solved crystal structure of rat PP5 (4JA9)^32^. Our SAXS/MS-based structural model is best compatible with a complex, where CeHsp90 and PPH-5 form a small interaction interface (~100 Å^2^) in addition to the binding interface with the C-terminal MEEVD peptide (Fig. 5A,B). Additional contacts in this area could originate from a negatively charged loop region in PPH-5 (residues 150–190) and positively charged regions in Hsp90 surrounding K510 and K551. This is consistent with the analytical ultracentrifugation data and suggests that the increased compactness is due to a combination of binding of the disordered Hsp90 C-terminus and PPH-5 in the central region of CeHsp90.

It is interesting to note that K510 and K551, while positioned close to each other in the 3D-structure, would originate each from a different CeHsp90 subunit in this model. This implies that PPH-5 could additionally affect the rates of dimer formation and dissociation. Thus, we tested whether PPH-5 is able to influence the dimerization properties of Hsp90 in an established yeast Hsp90 FRET system^33^. Dimerization kinetics can be measured after formation of FRET-pairs of differently labeled Hsp90-subunits. To induce dissociation, a large excess of unlabeled yHsp90 is added, which disrupts the FRET pair and uncovers the subunit dissociation kinetics. We recorded the dissociation kinetics in the absence and presence of PPH-5. Interestingly, the subunit exchange rate is indeed decreased in presence of PPH-5 (Fig. 5C), implying that PPH-5 does stabilize the dimeric state of yHsp90, supporting the arrangement of the two proteins as proposed in the SAXS/MS-models.

### The phosphatase is activated towards native clients by Hsp90 interaction

Finally, we aimed at understanding whether the cooperation between CeHsp90 and PPH-5 has relevance for the dephosphorylation of native Hsp90 client proteins. To this end, several putative substrates of the phosphatase or of Hsp90 were purified and analysed in dephosphorylation assays. As potential clients of Hsp90 and PPH-5 a fragment of the human glucocorticoid receptor (GR) was tested, and the transcription factors SMAD2 and SMAD4 as well as the protein-kinase ASK1 (Apoptosis signal-regulating kinase 1). All of them were shown to be dephosphorylated by PP5 *in vivo*^10^,^12^,^14^,^34^,^35^.

**Figure 6:**
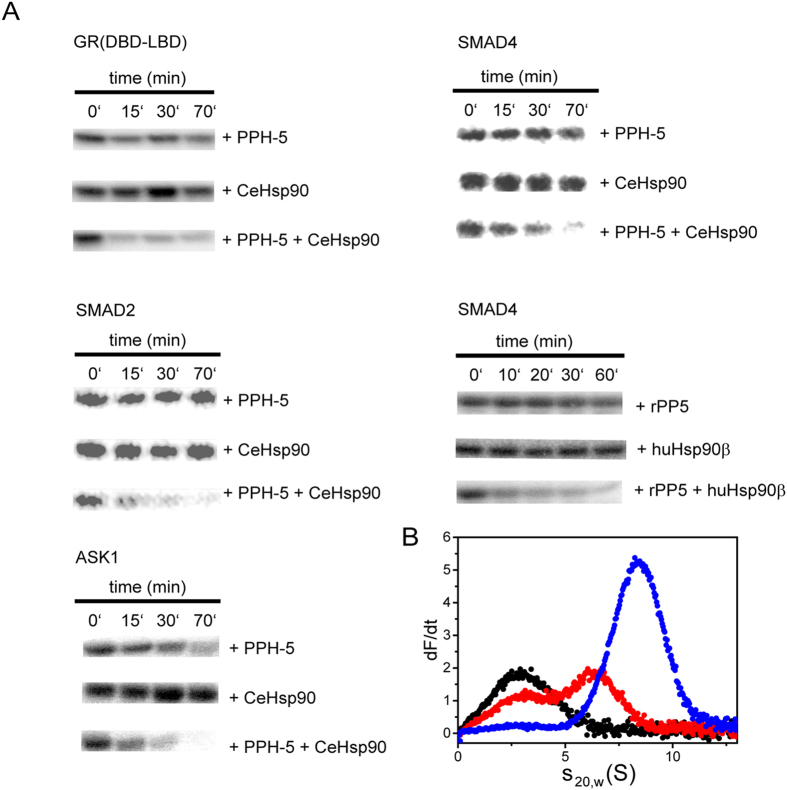
Hsp90 regulates substrate dephosphorylation of protein phosphatase 5. (**A**) Dephosphorylation of radioactive labelled substrates, which have been phosphorylated with [γ-^32^P] ATP by CaMKII. CeHsp90-dependent dephosphorylation of 9.5 μM GR-fragment, 9.5 μM SMAD2, 9.5 μM SMAD4 and 9.5 μM ASK1 by 3 μM PPH-5 in absence and presence of 3 μM CeHsp90. Dephosphorylation of 9.5 μM SMAD4 using 3 μM rPP5 or 3 μM human Hsp90β. For all tested substrates the addition of 3μM CeHsp90 or human Hsp90β alone did not lead to dephosphorylation. (**B**) Ternary complex formation between labeled *GR-LBD, CeHsp90 and PPH-5 was determined by analytical ultracentrifugation experiments. 400 nM of free *GR-LBD sediments with 2.8 S (black). Addition of 3 μM CeHsp90 increases the sedimentation coefficient to 6.5 S (red). A ternary complex is formed when adding 3 μM PPH-5 to the preformed complex with 8.4 S (blue). Addition of the phosphatase alone has only a weak effect on the sedimentation of the GR-LBD*.

The time-dependent dephosphorylation reaction for each substrate was recorded in presence of PPH-5 and in combination with CeHsp90. The dephosphorylation of GR, SMAD2 and SMAD4 (Fig. 6A) was only observable in the presence of a CeHsp90-PPH-5 complex, while PPH-5 alone was not active within the time range tested. In case of ASK1, the phosphatase could slightly dephosphorylate the kinase alone, but with a much higher activity in presence of CeHsp90 (Fig. 6A). Besides the nematode proteins, we tested this behaviour also for the mammalian system. Here likewise, the dephosphorylation of SMAD4 was only possible when rPP5 and human Hsp90β (huHsp90β) were supplemented together, confirming that this behaviour during client processing is conserved between the two species (Fig. 6A).

Given the striking increase in dephosphorylation activity, we tested whether we can detect ternary complexes of CeHsp90, PPH-5 and the client protein. To this end, we used an ultracentrifugation assay employing fluorescently labelled LBD of the GR (*GR-LBD). The LBD was found to sediment with a sedimentation coefficient of 2.8 ± 0.3 S in agreement with previous studies^29^. Like for huHsp90β the addition of CeHsp90 resulted in complex formation. The same is evident for nematode Hsp90 (Fig. 6B), which forms a complex with *GR-LBD at an s_20,w_. of 6.4 ± 0.3 S. Similar to experiments with human Hsp90, a certain amount of *GR-LBD still sedimented with 2.8 S, implying incomplete complex formation with rather low affinities. Addition of PPH-5 alone has no effect on the sedimentation of the *GR-LBD. However, addition of PPH-5 to CeHsp90-*GR-LBD complexes leads to formation of larger complexes as evident from the sharply increased s_20,w_, which is at 8.4 S ± 0.3 S for the ternary complex. Interestingly, much more *GR-LBD was also present in the large protein complexes diminishing the unbound *GR-LBD fraction to almost undetectable levels. This shows that both, Hsp90 and PPH-5, drag GR into large ternary protein complexes, which form with higher affinity compared to GR complexes with Hsp90 alone.

### C-terminal Hsp90 fragments stimulate in the absence of ternary complexes

**Figure 7:**
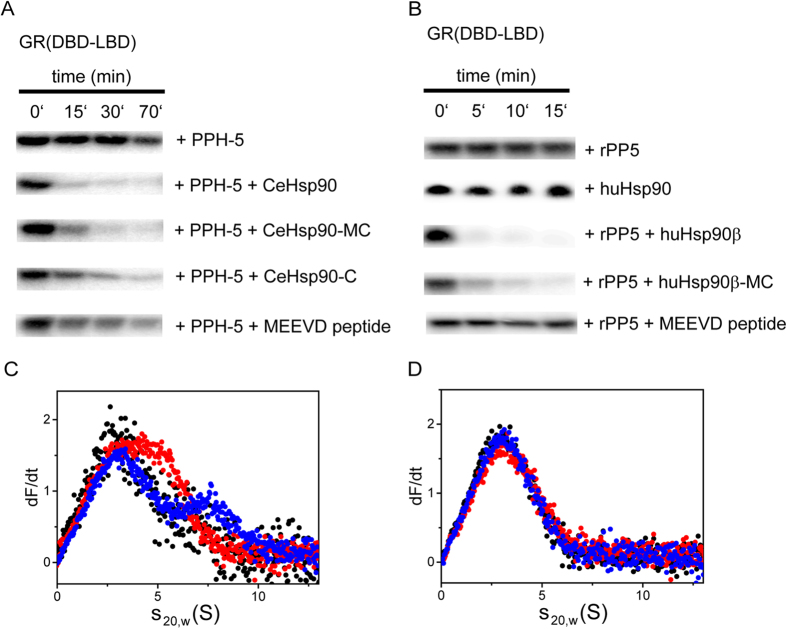
The MC interface of CeHsp90 is crucial for PPH-5 stimulation and substrate specificity. (**A**) Time-dependent dephosphorylation of GR(DBD-LBD) by PPH-5 in presence of different CeHsp90 constructs. 9.5 μM of ^32^P-phosphorylated GR(DBD-LBD) were treated with 3 μM PPH-5 alone and in combination with 3 μM CeHsp90, CeHsp90-MC, CeHsp90-C or the MEEVD peptide of CeHsp90. (**B**) The same experiment as in (**A**) was performed but this time the dephosphorylation activity of rPP5 was analysed in presence of human Hsp90β, human Hsp90β –MC and the MEEVD-containing peptide. Addition of Hsp90β alone did not lead to dephosphorylation (**C**) Ternary complex formation between labelled *GR-LBD (black), the CeHsp90-MC construct and PPH-5 was determined by analytical ultracentrifugation experiments. A complex is formed by adding the CeHsp90-MC (3 μM) construct to labelled *GR-LBD resulting in species with a sedimentation coefficient of 4.9 S (red) and a further increase by adding PPH-5 to 7.6 S (blue). Adding PPH-5 (3 μM) alone to labeled *GR-LBD leads to no complex formation (data not shown). (**D**) Complex formation between *GR-LBD, CeHsp90-C and PPH-5 was analysed using analytical ultracentrifugation experiments. 3 μM CeHsp90-C (red) forms no complex with labelled 400 nM GR-LBD (black) and after adding 3 μM PPH-5 also no peak shift to higher s_20,W_-values can be observed (blue).

Two different contributions of Hsp90 to the accelerated dephosphorylation of clients can now be envisioned. First, Hsp90 could act as an activator of the enzymatic activity of protein phosphatase 5. Secondly, Hsp90 could present clients to the phosphatase in ternary complexes, thereby increasing the efficiency of the substrate encounter. To differentiate between these possibilities we utilized different Hsp90-constructs and compared their contribution to stimulation and ternary complex formation with respect to *GR-LBD. Addition of the MEEVD peptide leads to dephosphorylation, but this stimulation of the phosphatase is weak compared to full-length CeHsp90 (Fig. 7A). The rate of dephosphorylation is higher in the presence of CeHsp90-C, but not nearly as high as in the presence of CeHsp90-MC. This comparison shows that the M-domain of CeHsp90 is required to obtain the high dephosphorylation rates observable with the full-length protein, although the C-terminal domain already displays a weak stimulatory potential.

We tested how this relates to the formation of ternary complexes with the individual Hsp90 constructs (Fig. 7C). In principle should all these fragments be much less prone to form ternary complexes with the GR-substrate than full-length Hsp90, as the N-terminal domain of Hsp90 is required for interaction with GR^29^. Indeed, we observe a weaker complex formation in analytical ultracentrifugation experiments with Hsp90-MC (Fig. 7B). Here, a significant part of the *GR-LBD sediments as unbound protein. Interestingly, in presence of PPH-5 a ternary complex with *GR-LBD can be observed. Nevertheless, the cooperative action between PPH-5 and Hsp90 to make high affinity complexes with GR-LBD depends on the N-terminal domain of Hsp90, highlighting the contribution of the Hsp90 N-domain to GR-LBD interactions^29^. Instead, Hsp90-C does not form ternary complexes in presence of PPH-5 (Fig. 7C), but still promotes the dephosphorylation of GR-LBD. Thus, this part of the stimulation apparently originates from direct effects on PPH-5’s enzymatic turnover, like the limited effects observable with the MEEVD-containing peptide.

To test the conservation of these results, we analysed the dephosphorylation of the GR-fragment with similar combinations of rPP5 and huHsp90β (Fig. 7B,D). Here, the huHsp90β-MC also stimulates the dephospharylation by rPP5. The MEEVD-containing peptide does not stimulate the rPP5 protein. Thus, although the MC-fragment of Hsp90 does not contain the N-domain required for full GR binding, it is able to stimulate the phosphatase activity in both systems. These results suggest that some basal activity originates from the stimulation of the phosphatase activity by regions in the M- and C-domains, but these effects may be further substantiated by the close arrangement of Hsp90, client protein and phosphatase in ternary complexes.

## Discussion

The Hsp90-associated protein phosphatase 5 has been shown to be a member of Hsp90-complexes with the glucocorticoid receptor^10^,^36^. However, the functional aspects of this interaction and the structural organization of this complex are mostly unknown. Here, we addressed the structural orientation of the Hsp90-phosphatase complex and the correlation with its dephosphorylating activity.

The activation Hsp90 imposes on PP5 was discovered more than a decade ago^37^ and attributed to the C-terminal MEEVD-motif of the chaperone^6^,^38^. Instead of the plain interaction between the MEEVD-peptide of Hsp90 and the TPR-domain of the phosphatase, we find a second interaction site. This site is located within the C-terminal and middle domain of Hsp90 and further increases the activity of the phosphatase. The structural analysis based on SAXS, analytical ultracentrifugation and Xlinked mass spectrometry shows that the binding arrangement of the phosphatase, which is usually tightly bound at the TPR/MEEVD interaction site^9^, is flexible enough to promote interactions with other parts of the chaperone protein. It is conceivable that the alignment of the phosphatase domain with the Hsp90 protein might allow a cooperative work on client proteins, leading to a specific effect on the dephosphorylating activity beyond that of the individual proteins.

Indeed, we could proof this assumption for the native client GR. Here, no dephosphorylating activity is observed in the absence of Hsp90, but increased dephosphorylation is displayed after addition of C-terminal or longer fragments of Hsp90. Apparently, two effects are relevant in these complexes. Firstly, the complexes of the protein phosphatase with C-terminal Hsp90-fragments show a higher dephosphorylation activity also towards native substrates, which originates from a stimulation of the protein phosphatase 5 by Hsp90-C. A stronger effect is observed, if the middle domain of Hsp90 is additionally present. This can be observed for the peptide substrate KRpTIRR and for the native client GR. It implies that once the full interaction region between PPH-5 and Hsp90 is present the highest stimulation of the phosphatase domain is obtained. This may originate from the previously reported release of the autoinhibition of protein phosphatase 5 by the MEEVD-peptide^38^,^39^ and additional stimulation originating from the direct contact between Hsp90 domains and the phosphatase domain.

Beyond stimulating the phosphatase activity, the binding of native substrates, like the glucocorticoid receptor, appears to be cooperative in the presence of the full-length Hsp90, leading potentially to a very specific client protein orientation in these complexes. Such mechanisms may safeguard that dephosphorylation is only performed on client proteins, which encounter ternary complexes with Hsp90 and the phosphatase and not on any other proteins in the cell. Thus, it is important to learn that further contacts between these two proteins can exist, which may be responsible for such regulation. While the uncovered crosslinking sites show that the association of protein phosphatase 5 with more N-terminal parts of Hsp90 is in principle possible, the exact mechanisms of the regulation remain to be uncovered. Nevertheless, the described interaction region may prove to be relevant also for the functional aspects of this interaction, which require the cooperative action of client binding interfaces, PP5-binding interfaces on Hsp90 and probably client-binding interfaces on PPH-5 to form the ternary complexes observed in the presence of the native client GR.

It is also important to note that many more client proteins for protein phosphatase 5 and Hsp90 are getting uncovered, which include protein kinases and other transcription factors. Given that these proteins have widely different structures it is to be assumed that the formation of Hsp90-protein phosphatase 5 client protein complexes require a certain flexibility also on side of the chaperone system and its binding properties.

## Methods

### Protein expression and purification

The pET28b plasmid was used as expression vector for proteins harbouring an N-terminal His_6_-tag. Proteins were expressed in the BL21-CodonPlus(DE3)-RIL strain. After growth at 37 °C to an OD_600_ of 0.6 cells were induced by addition of 1 mM IPTG. Cells were incubated overnight and harvested at 6000 rpm and 4 °C for 15 min. Cell pellets were resuspended in Ni-NTA equilibration buffer (40 mM HEPES/KOH, pH 7.5, 150 mM KCl; 1 mM DTT) and mechanically disrupted. The supernatant was applied onto a HisTrap FF 5ml Ni-NTA column and elution was induced by increasing imidazole concentrations. After dialysis against 40 mM HEPES/KOH, pH 7.5, 20 mM KCl, 1 mM DTT, the protein was applied onto a Resource Q column and eluted in a salt gradient. Pure fractions were further purified on a Superdex 75/200 gel filtration column. The protein was finally dialyzed against storage buffer (40 mM HEPES/KOH, pH7.5, 20/150 mM KCl, 1 mM DTT), concentrated and frozen at −80 °C. Protein purity and identity was assessed by MALDI-TOF mass spectrometry (Bruker, Bremen, Germany) and SDS-PAGE to be >95% pure. GR expression and purification was performed as described previously^29^.

### Phosphatase assays with p-nitrophenyl phosphate

p-nitrophenyl phosphate (pNPP) was used as substrate in phosphatase assays. The phosphate group of pNPP can be cleaved off by phosphatases releasing p-nitrophenol. This reaction was recorded at 410 nm (ε = 15100 M^−1^ cm^−1^). The assay was measured in 40 mM HEPES/KOH, 20 mM KCl, 5 mM MnCl_2_, 1 mM DTT, pH 7.5 and 0–60 mM pNPP at 20 °C if not indicated otherwise. The phosphatase concentration was 100 nM. If indicated, Hsp90 (3 μM), Hsp90-C (C-terminal domain, 3 μM)) or the C-terminal peptide of nematode Hsp90 (AEEDASRMEEVD, 60 μM) was supplemented in the assays.

### Peptide dephosphorylation assays

The EnzChek Phosphate Assay Kit (Invitrogen, La Jolla, USA) was used according to the manufacturer’s protocol. Briefly, free phosphate, originating from PPH-5 induced dephosphorylation of the substrate peptide KRpTIRR, was detected. The reaction buffer provided with the assay kit was supplemented with 5 mM MnCl_2_. Measurements were performed at 20 °C. 50 μM of the peptide were incubated with 250 nM PPH-5 alone and in presence of 3 μM Hsp90, 3 μM Hsp90-MC 3 μM Hsp90-C or up to 60 μM of the Hsp90 MEEVD-containing peptide.

### Fluorescence-labelling of GR-LBD

0.1 mg of cysteine reactive Alexa 488-maleimide (Invitrogen) was dissolved in DMSO and added to 1 mg of protein with a final DMSO concentration of 1%. The reaction was incubated at RT for 1 h and stopped by adding 100 mM DTT. Free label was separated from labelled protein using a Superdex 75 HR size exclusion column, pre-equilibrated with 40 mM HEPES, 150 mM KCl, 1 mM DTT, pH 7.5. The elution was detected by UV absorption and fluorescence at the excitation wavelength of 488 nm and the emission wavelength of 515 nm. Eluted protein was aliquoted and stored at −80 °C.

### Analytical ultracentrifugation (aUC)

Binding of PPH-5 to Hsp90 and Hsp90-MC was analysed by analytical ultracentrifugation using the UV/VIS optical system of the Beckman XL-A centrifuge. At a constant Hsp90 concentration of 4 μM, different concentrations of PPH-5 were added, leading to the formation of Hsp90 complexes of higher s_20,w_-value and accumulation of free PPH-5. These concentrations were determined with the Sedfit software^40^. Concentration values were fit to an equilibrium model describing sequential binding of the first and second PPH-5 molecule to Hsp90^41^ using the absorption readings to independently verify the PPH-5 and Hsp90 concentrations during the fitting procedure. The absorption readings of Hsp90 and of the saturated Hsp90-PPH-5 complex were used to confirm the binding stoichiometry in the model. The equations describing the whole system based on the floating parameters M_t_ (total Hsp90), correction factor for L_t_ (total PPH-5), Kd_1_, Kd_2_ and the fixed parameters ε_Hsp90, 280nm_ = 112000 m−1 cm−1, ε_PPH-5, 280nm_ = 51000 m−1 cm−1, were implemented in Microsoft Visual Studio 2013 and linked to Origin as dll-file. While it is clear from the data that two PPH-5 molecules bind to the Hsp90 dimer, the resolution and data quality is not sufficient to obtain separate values for Kd_1_ and Kd_2_. The correlation between f/f0 and the molecular weight of the proteins and complexes was determined with the help of SedFit.

Binding of GR-LBD to the chaperones was analysed by analytical ultracentrifugation coupled with fluorescence detection as described before^29^,^42^. Experiments were performed in a Beckman ProteomeLab XL-A analytical ultracentrifuge (Beckman Coulter, Brea, USA) equipped with an Aviv AU-FDS detector (Aviv Biomedical, Lakewood, USA). AUC runs were performed in 40 mM HEPES/KOH, pH 7.5, 20 mM KCl, 1 mM DTT. The concentration of GR-LBD was 400 nM and the concentration of Hsp90 and the phosphatase varied as indicated. Data analysis was performed using a dc/dt approach according to Stafford^43^,^44^. The dc/dt plots were fit to gaussian functions in order to obtain the s_20,w_ values of the respective species as described previously^42^.

### Crosslinking experiments and mass spectrometry analysis

Proteins were crosslinked in 40 mM Hepes, 150 mM KCl, 1 mM DTT, pH 7.5 for seven minutes at RT, using isotope labelled di-sulfo-succinimidyl-glutarate (DSSG-H6/D6) (Creative Molecules Inc.) in a 40-fold excess over protein. The crosslinking reaction was stopped by addition of 1 × Laemmli buffer. Samples were analysed on 4–12% gradient gels (Serva Electrophoresis GmbH) and bands representing crosslinked species were excised. Proteins were extracted according to previously established protocols^45^. The final peptide mixtures were analysed by a LTQ Orbitrap XL (Thermo Scientific) using ESI (Nanospray). Peptide mixtures were separated by an Acclaim PepMap RSLC C18 trap column (75 μm × 150 mM, C18, 2 μm, 100 Å; Thermo Scientific) on a HPLC Ultimate 3000 RSLCnano System (Dionex) before being analysed by a LTQ Orbitrap XL instrument. Peptides were eluted by applying a linear gradient from 5 to 35% acetonitrile with 0.1% formate. MS spectra were acquired in the Orbitrap with a resolution of 60,000 in a m/z-range from 300–2000 Da. The 3 most intense peaks (charge range>+3) in one full scan MS spectrum were selected for CID- and HCD-fragmentation (CID-fragmentation: collision energy = 35 V, HCD-fragmentation: collision energy = 40 V).

Two approaches were used to identify the crosslinked peptides in the three recorded data sets. Data were analysed with MaxQuant to obtain peak lists containing intensity values and elution times^46^. From that point a procedure (xMass) written in the integrated development environment Code::Blocks was used to specifically search for cross-linked peptides. To this end, all MaxQuant-derived peaks were imported and xMass determined all potential pairs of peaks, which deviated in mass by 6.043 Da and could be assigned to a potential X-linked product from the simulated product library. The threshold was set to 15 ppm, 2 miscleavages were allowed in each crosslinked peptide. Fragment spectra for MS/MS-analysis were obtained from the .raw files with the program RAXPORT^47^. If calculated peak lists for candidates showed sufficient overlap with the MS/MS spectra (set to >15 matches for two peptides containing products and >8 matches for single peptides), this peak pair was maintained as potential hit. Final assignment as hit required that the crosslinked lysine was not the cleavage site and each peptide contained more than three amino acids.

Identification of cross-linked peptides was in parallel performed with help of the program pLink^48^. The following pLink search parameters were used: mass tolerance 15 ppm, fragment mass tolerance: 15 ppm, cross-linker DSSG-H6/D6 (cross-linking sites K and protein N-terminus, isotope shift 6.04368 Da, xlink mass shift 96.02059 Da, monolink mass shift 114.03115 Da), variable modification: Oxidation (Met) – 15.995 Da, peptide length minimum 4 amino acids and maximum 100 amino acids, enzyme: trypsin with max. 2 missed cleavage sites. As relevant interpeptide crosslinks we used in particular those, which were verified by both approaches.

### Radioactive dephosphorylation assay

Radioactive phosphorylated proteins were obtained as described before^49^. 9.5 μM of SMAD2, SMAD4, and the DBD (DNA binding domain) – LBD (ligand binding domain) of the human glucocorticoid receptor were phosphorylated with [γ-^32^P]ATP using 0.1 mU Calmodulin-dependent protein kinase 2 (BioLabs) at 30 °C for 120 min. Remaining ATP was hydrolysed by addition of apyrase (BioLabs) for 30 min at 20 °C. Phosphorylated proteins were then incubated with 3 μM of C. elegans or human Hsp90 (huHsp90), one of its fragments or the MEEVD-containing peptide and 3 μM PPH-5 at 20 °C. After various time points, samples were taken and dephosphorylation was stopped by addition of Laemmli buffer and heating to 95 °C. The relative degree of phosphorylation was analysed by SDS-PAGE followed by phosphoimaging on a Typhoon 9200 Phosphoimager (Amersham Biosciences).

### Small-angle X-ray Scattering measurements and data analysis

SAXS data for solutions of C. elegans Hsp90 (CeHsp90), PPH-5, and the CeHsp90-PPH-5 complex were recorded on an in-house SAXS instrument (SAXSess mc2, Anton Paar, Graz, Austria) equipped with a Kratky camera, a sealed X-ray tube source and a two-dimensional Princeton Instruments PI•SCX:4300 (Roper Scientific) CCD detector. The scattering patterns were measured with a 90/180 min. exposure time (540/1080 frames, each 10 seconds) for several solute concentrations in the range from 0.8 to 6.3 mg/ml. Radiation damage was excluded based on a comparison of individual frames of the 90/180-min. exposures, where no changes were detected. A range of momentum transfer of 0.012 < s < 0.63 Å^−1^ was covered (s = 4π sin(θ)/λ, where 2θ is the scattering angle and λ = 1.5 Å is the X-ray wavelength). All SAXS data were analysed with the package ATSAS (version 2.5). The data were processed with the SAXSQuant software (version 3.9), and desmeared using the programs GNOM and GIFT. The forward scattering, I(0), the radius of gyration, R_g_, the maximum dimension, D_max_, and the inter-atomic distance distribution functions, (P(R)), were computed with the program GNOM. The masses of the solutes were evaluated by comparison of the forward scattering intensity with that of a human serum albumin reference solution (molecular mass 69 kDa) and using the Porod volume. The structures of Hsp90 in complex with PPH-5 were modelled using the program CORAL. Input were the homology models of Hsp90-NM, and -C domains (based on PDB 2CG9, determined using Swiss-Model)^50^, a homology model of PPH-5 in complex with the Hsp90-MEEVD peptide (based on PDB 2BUG and 4JA7, determined using Swiss-Model, PPH-5 residues 27–493 and Hsp90 residues 698–702), SAXS data for the Hsp90-PPH-5 complex, and pair-wise MS cross-linking restraints defining the maximal Cα-Cα distance of 25 Å (given by the length of the MS cross-linker and the length of the lysine side chain) between residues in PPH-5 and Hsp90 (K205-K510, K205-K551, K206-K585). The dimerization interface of the Hsp90-C domain was maintained. Throughout the calculations the following residues were defined flexible and randomized: i) 29 residues in the N-terminus of Hsp90, ii) a short linker connecting the Hsp90-M and -C domains (residues 518–523), iii) the flexible C-terminus of Hsp90 connecting Hsp90-C and the MEEVD peptide (residues 668–697), and iv) 26 residues in the flexible N-terminus of PPH-5. A total of 50 structures were calculated, and the best structures based on the fit to the experimental data selected to prepare the figures.

Fluorescence resonance energy transfer measurements - FRET measurements were performed as described^33^. Yeast Hsp90 (yHsp90) was labelled at an engineered Cys61 in its N-terminal domain. As an acceptor the fluorescent dye ATTO-550-maleimide (ATTO-Tec GmbH, Siegen, Germany) was attached; as donor ATTO-488-maleimide was coupled to yHsp90. Analyses were carried out in a 40 mM HEPES/KOH, pH 7.5, 20 mM KCl and 5 mM MgCl_2_ buffer at 30 °C in a Fluoromax 3 fluorescence spectrophotometer (Horiba JobinYvon, Kyoto, Japan). Fluorescently labeled proteins were used at 200 nM each and kinetics measured in absence and presence of 6 μM PPH-5. Subunit exchange was initiated by adding a ten-fold excess of unlabelled yHsp90.

## Additional Information

**How to cite this article**: Haslbeck, V. *et al.* The activity of protein phosphatase 5 towards native clients is modulated by the middle- and C-terminal domains of Hsp90. *Sci. Rep.* **5**, 17058; doi: 10.1038/srep17058 (2015).

## Figures and Tables

**Table 1:** SAXS data and analysis.

Sample	Stoichiometry	R_g_ [Å]	D_max_ [Å]	Molecular mass [kDa]
Hsp90	2	59.1 ± 1.0	195	170
PPH-5	1	29.2 ± 0.3	100	63
Hsp90-PPH-5	2:1	65.3 ± 0.8	240	240
	2:2	61.9 ± 0.8	240	209

The apparent molecular mass and R_g_ is shown for CeHsp90 and PPH-5 alone and in complex with each other. The molecular mass was determined from the Porod volume.

**Table 2:** Crosslinked peptides of PPH-5 and CeHsp90 identified by xMass and pLink.

Peptide 1	Peptide 2	Type	Lys1*	Lys2*	Experiment--Elution step	MS2-Scan	Hit Number in Scan	Hit% Spectral Intensity	Hit% of peaks	Independent analysis of Experiment 3
					xMass (best MS2 scan)					pLink
PPH-5										
EkAGMIKDEANQFFK	—	1	28	0	3—1	1764	44	37	13	**√√**
ELYGSALEDADNAIAIDPSYVKGFYR		1	97	0						**√**
FkKALTDYQAVVK	—	1	113	0	1—1	1318	15	43	25	
FKkALTDYQAVVK	—	1	114	0	1—1	1318	15	43	25	
ALTDYQAVVkVCPNDKDAR	—	1	124	0	3—1	1548	33	11	11	**√√√**
RQkFEAAISTDHDK	—	1	147	0	3—2	923	46	50	20	
QkFEAAISTDHDKK	—	1	147	0	3—1	939	56	56	21	
QkFEAAISTDHDK	—	1	147	0	3—2	1073	44	51	30	**√√√**
QKFEAAISTDHDkK	—	1	158	0	2—5	886	16	35	16	
FEAAISTDHDkK	—	1	158	0	3—1	960	28	52	20	**√√**
LEDKITkEFVLQLIK	—	1	187	0	3—1	2465	21	18	11	**√√√√**
ERE**K**EVEDEEAVEAK	—	1	216	0						**√**
EFVLQLI**K**TF**K**NQQK	—	2	195	198						**√√**
SYEDEkEK	AGMIkDEANQFFK	3	26	33	3—2	1608	59	29	21	**√√√√**
CeHsp90										
ELISNASDALDkIR	—	1	46	0	2—5	1898	30	50	34	
ADLVNNLGTIAkSGTK	—	1	100	0	2—5	1756	34	56	30	
KIkEIVK	—	1	192	0	2—2	876	12	21	20	
kHSQFIGYPIK	—	1	197	0	3—1	1296	30	56	20	**√√√√√**
HSQFIGYPIkLVVEK	—	1	207	0	2—5	1997	45	39	26	
EREkEVEDEEAVEAK	—	1	216	0	3—1	952	58	46	24	
KFYEQFGkNLK	—	1	414	0	2—5	1367	18	26	16	
VIKDILEkK	—	1	551	0	2—1	1205	9	20	14	
KVEkVGVSNR	—	1	555	0	1—1	563	26	65	27	**√**
kHLEINPDHAIMK	—	1	602	0	3—3	1202	74	53	19	**√√√**
VEVDKNDkTVK	—	1	627	0	1—1	634	10	47	20	
EMLQQSkILKVIR		2	378	381						**√**
IkEIVK	EMLQQSkILK	3	192	378	3—3	1039	43	28	11	
EIVkK	YFEDEELNkTKPIWTR	3	196	263	3—3	1593	31	9	10	
EMLQQSkILK	kNLVK	3	378	385	1—1	1130	25	52	18	**√√√√√**
ILKVIR	kNLVK	3	381	385						**√√√√√**
kNLVK	kLSDFLR	3	385	429						**√√√√**
VIkDILEK	KVEkVGVSNR	3	546	555	1—1	1080	18	31	13	
VIkDILEK	DSSTMGYMAAkK	3	546	601	3—1	1625	49	13	13	
DILEkK	DSSTMGYMAAkK	3	551	601	3—1	1451	34	36	18	
CeHsp90 and PPH-5										
kLVSVTK	LHkK	4	510	205	1—1	521	23	49	17	**√√√√**
DILEkK	LHkK	4	551	205	3—3	724	19	48	15	**√√√√**
IMkAQALR	kYAFK	4	585	206	3—5	2247	16	25	17	

Shown are the crosslinked products detected by mass spectrometry using two different search algorithms, xMass and pLink. The crosslinking type refers to the mode of attachment of the crosslinker H_6_/D_6_-DSSG: 1) Single-sided attached crosslinker, water-quenched on the other side. 2) Linear peptide with both sides of the crosslinker attached. 3) Two peptides of the same protein crosslinked. 4) Two peptides originating from both proteins crosslinked. *) amino acid positions are given without His-tag (otherwise +23 for PPH-5 and +21 for CeHsp90). The second number refers to the elution step, in the cases, where ion exchange zip-tips were used prior to the mass spectrometry. MS2 Scan mentions the scan, which gave the most hits for the particular peptide. Exclusion criteria of xMass were (<10% of the peaks in the MS/MS spectrum, <4 amino acids per peptide, <15 Hits in MS/MS per crosslinked peptides (Type3, 4) or <8 Hits in MS/MS per attached peptide (Type 1, 2), modified lysine as cutting site). The parallel analysis refers to usage of the pLink software. Checkmarks correspond to the number of elution steps, which contained the respective peptide.
